# Glibenclamide, a diabetic drug, prevents acute radiation-induced liver injury of mice via up-regulating intracellular ROS and subsequently activating Akt–NF-κB pathway

**DOI:** 10.18632/oncotarget.16501

**Published:** 2017-03-23

**Authors:** Hu Liu, Shichao Wang, Zhao Wu, Ziyun Huang, Wei you Chen, Yanyong Yang, Jianguo Cui, Cong Liu, Hainan Zhao, Jiaming Guo, Pei Zhang, Fu Gao, Bailong Li, Jianming Cai

**Affiliations:** ^1^ Department of Radiation Medicine, Faculty of Naval Medicine, Second Military Medical University, Shanghai, China; ^2^ Fifth Cadet Battalion of Naval Medicine Department, Second Military Medical University, Shanghai, China

**Keywords:** acute radiation-induced liver injury, reactive oxygen species (ROS), ATP-sensitive potassium channel (KATP), membrane potential (MP), glibenclamide

## Abstract

**Background:**

Acute radiation-induced liver injury is a limitation for hepatoma radiotherapy. Come so far the clinical treatments are insufficient. The effective, specific, low toxicity and novel drugs are in powerful need. Glibenclamide is a common hypoglycemic. Some studies have revealed its relation with intracellular reactive oxygen species, the crucial mediator to radiation injury. This study is aimed to investigate if glibenclamide could act on the acute radiation-induced liver injury.

**Results:**

Glibenclamide mitigated acute radiation-induced liver injury of mice, indicating as regression of hepatocellular edema, reduction of hepatic sinusoid, decline in serum ALP level and reduction of hepatocellular apoptosis. Pretreatment of glibenclamide reduced the radiosensitivity of NCTC-1469 cells. In mechanism, glibenclamide elevated cells membrane potential to up-regulate intracellular reactive oxygen species. The increased reactive oxygen species subsequently activated Akt–NF-κB pathway to promote survival of irradiated cells.

**Methods:**

BALB/C male mice were intraperitoneal injected with glibenclamide 1 hour before hepatic irradiation. At designed time points the livers were taken to make histological study and bloods were collected to measure serum transaminase. With/without glibenclamide pretreatment the irradiated NCTC-1469 cells were tested apoptosis, viability and proliferation. By western blotting the involved molecules were detected.

**Conclusions:**

Glibenclamide, prevents acute radiation-induced liver injury of mice via up-regulating intracellular reactive oxygen species and subsequently activating Akt–NF-κB pathway.

## INTRODUCTION

Radiotherapy is an important treatment for hepatic carcinoma. Especially the stereotactic body radiation therapy (SBRT) and radioembolization make the radiotherapy for hepatic carcinoma more prevalent [1, 2]. However inevitable radiation injury to liver is still the largest challenge to radiotherapy [3]. Radiation-induced liver injury is a common radiation injury in liver, which usually occurs in the early stage of radiation and is characterized by anicteric ascites and elevation in liver enzymes [4].

Usually acute radiation-induced liver injury could remit itself within several weeks, but sometimes it could deteriorate and develop to lethal liver failure [5]. Come so far the clinical therapeutics for acute radiation-induced liver injury mainly includes steroid and some antioxidant. Side effects and lack of specificity are the limitations of these drugs. Therefore effective, specific, less toxicity and novel drug is in powerful need.

In mechanism Reactive oxygen species (ROS) is generally believed as the crucial mediator to radiation injury, which subsequently damages biomacromolecules such as DNA and proteins [6]. Thus targeting ROS is considered to be an effective strategy for ameliorating radiation injury.

Glibenclamide is a common drug for diabetes mellitus since its production in 1957 [7]. Glibenclamide could block the ATP-sensitive potassium channel (K_ATP_ channel) to induce the Ca^2+^ influx and subsequently promote release of insulin so that regulate the blood sugar level [7]. Recent studies have demonstrated that K_ATP_ channel, especially the mitochondrial membrane ATP-sensitive potassium channel (mK_ATP_ channel) is associated with generation of intracellular ROS and that is to say, glibenclamide may play a regulated role in generation of intracellular ROS [8, 9]. However its exact effect remains controversial. Some studies showed an elevated ROS from glibenclamide [10] but some reports opposites this conclusion [11]. At all events, by targeting K_ATP_ channel glibenclamide indeed regulates intracellular ROS level.

As liver is the one of the few tissues that highly expressed K_ATP_ channel, then we hypothesized that glibenclamide could influence acute radiation-induced liver injury by regulating ROS. Herein we made a study to investigate this effect and further to lucubrate involved mechanism. More meaningfully, as glibenclamide is a common hypoglycemic, its low toxicity is explicit. Hence if glibenclamide influence acute radiation-induced liver injury then it would be potential in clinical application.

## RESULTS

### Establishment of acute radiation-induced liver injury model of mice

**Figure 1:**
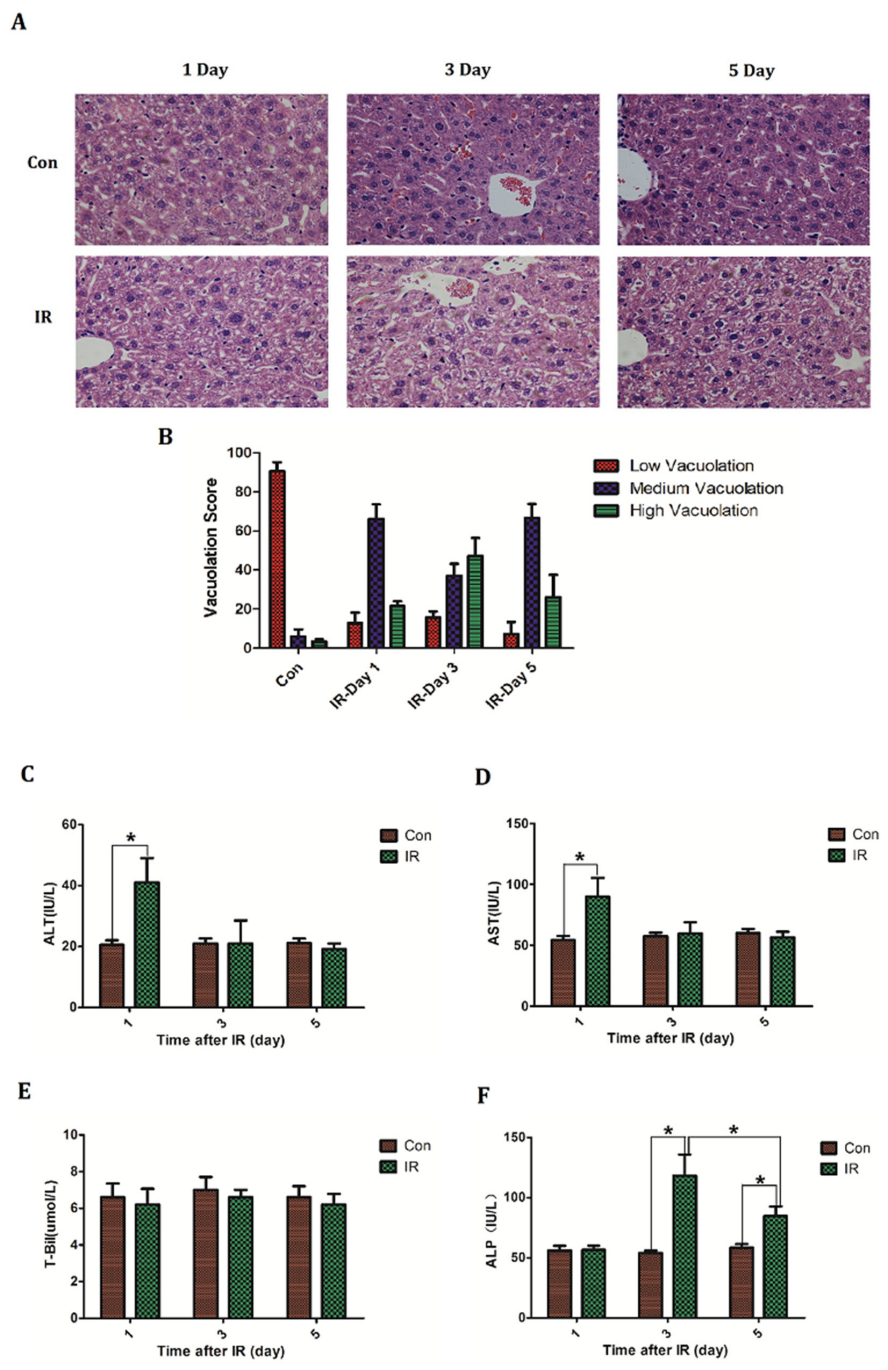
The establishment of acute radiation-induced liver injury model of mice BABL/C mice received hepatic 30Gy of irradiation. On the first, third and fifth day after irradiation the livers were taken to **(A, B)** make histologic evaluation by H&E staining (n=5) (micrographs in A; quantified in B); Bloods were collected to measure **(C)** ALT level, **(D)** AST level, **(E)** T-Bil level and **(F)** ALP level (n=5). Magnification × 200. Data were presented as mean± SD. *P<0.05.

Hepatic regions of BABL/C mice were irradiated at a single dose of 30 Gy. At designed time (Day1, 3, 5) livers were taken to make histopathological study by H&E staining. Bloods were collected to measure serum glutamic-pyruvic transaminase (ALT), glutamic oxalacetic transaminase (AST), total bilirubin (T-Bil) and alkaline phosphatase (ALP) level respectively. Histologically there was extensive hepatocellular edema surrounding sublobular veins and local erythrocyte exudation under venous endodermis after irradiation, which was characterized by poorly defined cytoplasmic space and clear space within hepatocytes. Notably the most severe edema occurred on the third day after irradiation (Figure 1A, 1B). Serum levels of transaminases are common index of liver injury and then the serum ALT, AST level were detected after irradiation. Besides, total bilirubin (T-Bil) and alkaline phosphatase (ALP) were also measured to assess the liver function. Results demonstrated a slight elevation of ALT (Control group: 20.6±3.2 *VS* IR group: 41±18.0; *P*=0.037) and AST (Control group: 54.4±7.3 *VS* IR group: 91.8±33.9; *P*=0.042) on the first day after irradiation, and thereafter back to normal level (Figure 1C, 1D). Serum T-Bil level didn't reveal significantly change after irradiation (Figure 1E). However the serum ALP level rose after irradiation and peaked (Control group: 54±4.6 *VS* IR group: 118.2±39.2; *P*=0.0066) on the third day (Figure 1F). These results are in line with previous studies concerning acute radiation-induced liver disease which described an isolated elevation in alkaline phosphatase out of proportion to other liver enzymes [3–4].

### Glibenclamide mitigated acute radiation-induced liver injury of mice

**Figure 2:**
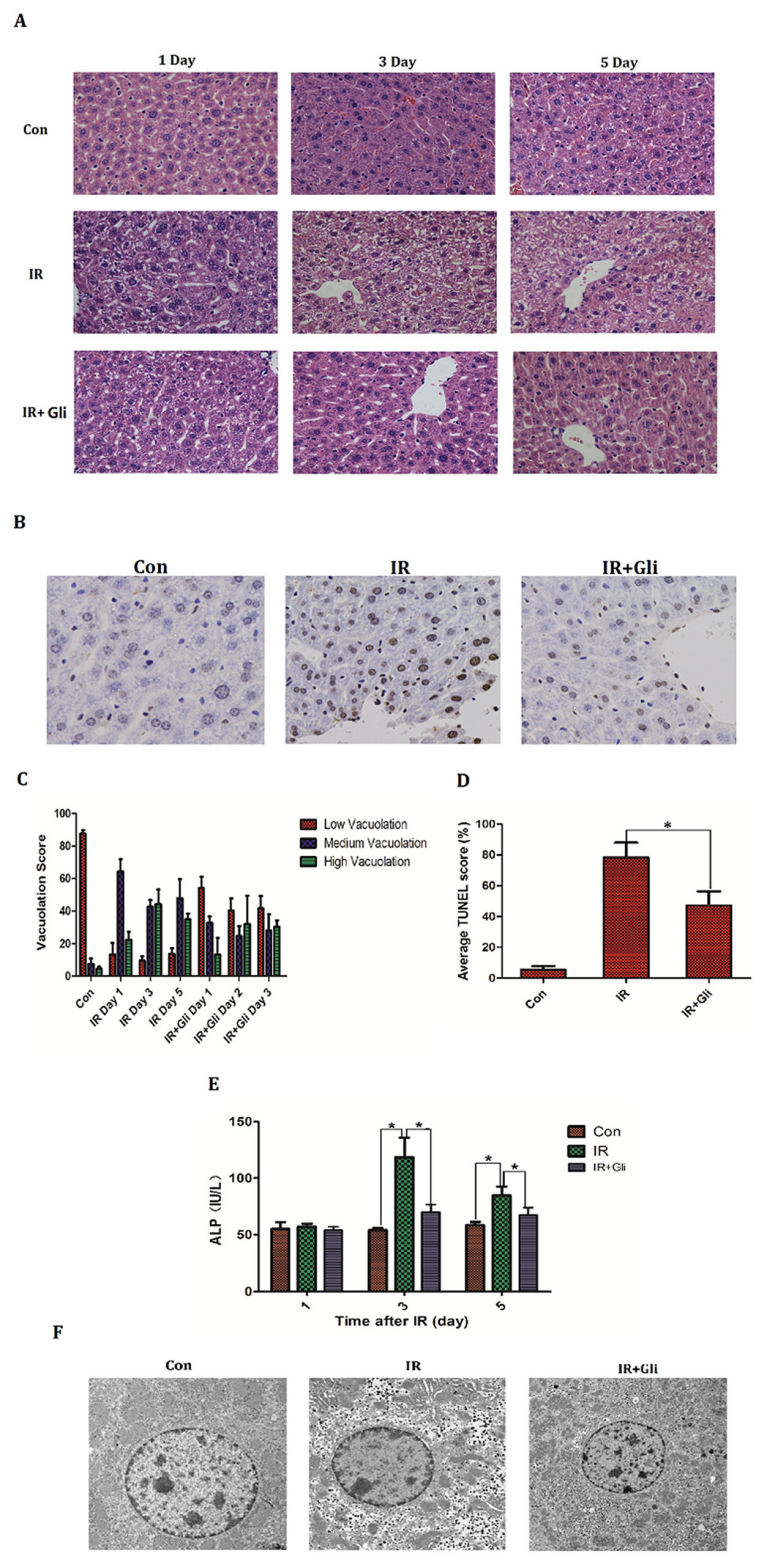
Glibenclamide remitted acute radiation-induced liver injury of mice 1 hour before irradiation, BABL/C mice received intraperitoneal injection of glibenclamide (10mg/kg). On the first, third and fifth day the livers of mice (n=5) were taken to **(A, C)** make histopathological analysis by H&E staining (micrographs in A; quantified in C; Magnification x 200) and blood were collected to **(E)** measure serum ALP level. The mice livers taken on Day 3 (n=5) were detected apoptosis by **(B, D)** dUTP nick-end labeling (TUNEL) assay (micrographs in B; quantified in D; Magnification × 400) and **(F)** electron microscopic observation. Arrows represent cytopathic sites. Data were presented as mean± SD. *P<0.05.

1 hour before irradiation, BABL/C mice received intraperitoneal injection of glibenclamide (10mg/kg). On the designed time (Day 1, 3, 5) mice livers were taken to make histopathological analysis by H&E staining and bloods were collected to measure serum ALP level. Besides the specimens taken on Day 3 were detected apoptosis by dUTP nick-end labeling (TUNEL) assay and electron microscopic scan. As a result, the advanced injection of glibenclamide mitigated acute radiation-induced liver injury, presenting as regression of hepatocellular vacuolation (Figure 2A, 2C) and decline in ALP level (Figure 2E). TUNEL was expressed significantly more often in the IR group (Control group: 5.3±2.5% *VS* IR group: 78.3±9.5%; *P*<0.001) but decreased in the IR+Gli group (IR+Gli group: 47.3±9.1% *VS* IR group: 78.3±9.5%; *P*=0.015) (Figure 2B, 2D). With electron microscopic the irradiated hepatocyte presented as cytoplasmic degradation, increased lacuna of karyotheca and chromatic pyknosis. Meanwhile glibenclamide improved above lesion (Figure 2F).

### Glibenclamide reduced radiosensitivity of mice hepatocyte

**Figure 3:**
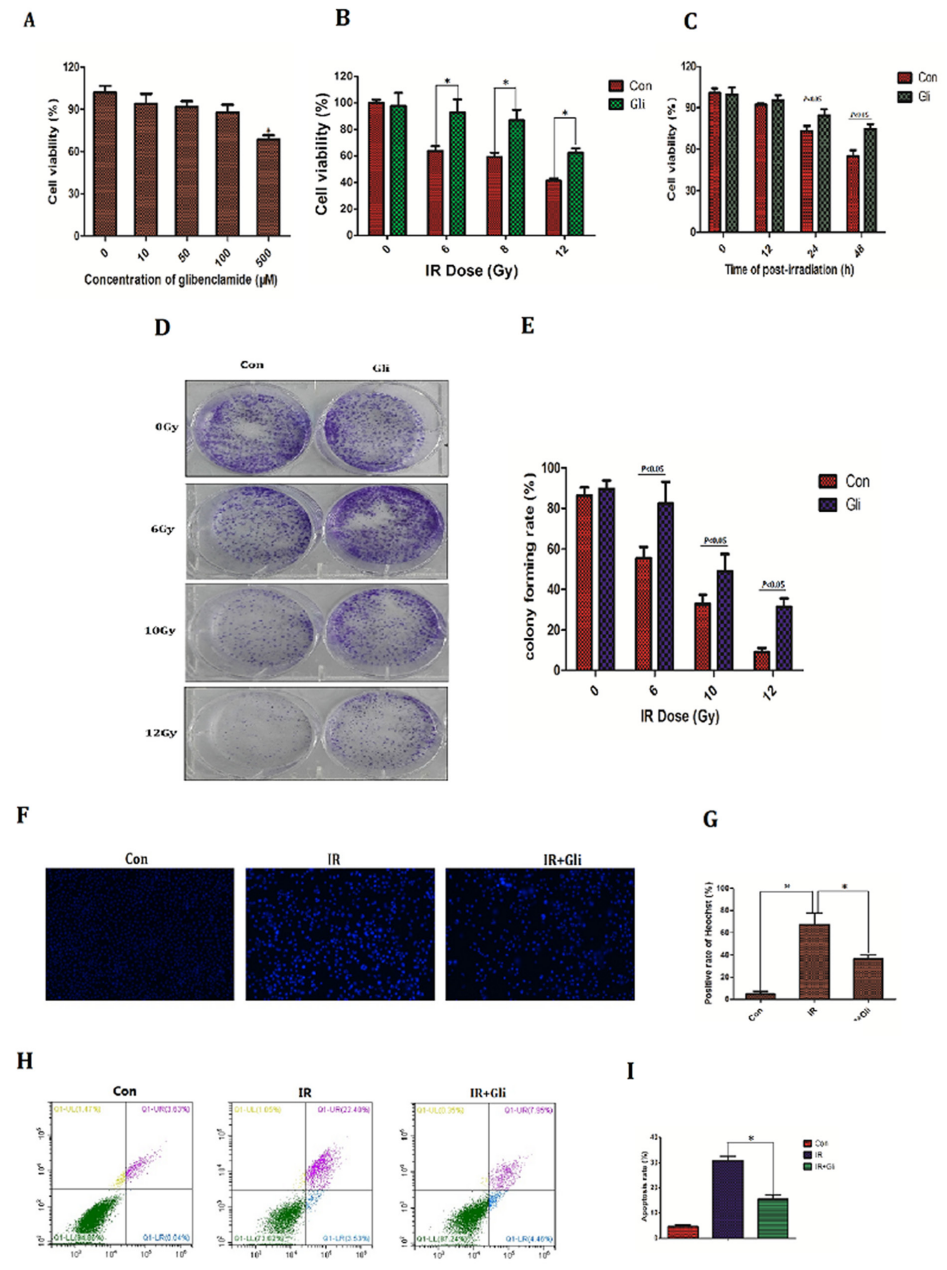
Glibenclamide reduced radiosensitivity of mice hepatocyte line **(A)** NCTC-1469 cells were treated with glibenclamide (100μM) for 1 hour. 24 hour later cells viability was tested by CCK-8 assay to evaluate the glibenclamide toxcity. Pre-treated with glibenclamide (100μM) for 1 hour, cells viability under **(B)** different IR dose and **(C)** different time point were determined. **(D)** 10 days after radiation the clones were dyed with crystal violet and **(E)** were quantified. 48 hours after radiation cells were harvested to test apoptosis rate by Hoechst33342 staining (micrographs in **F**; quantified in **G**) and **(H, I)** flow cytometry. n=3, Data were presented as mean± SD. *P<0.05

Since the protective effect of glibenclamide on irradiated hepatic tissue had been verified, we next investigated if there is as well same effect on hepatocyte. The NCTC-1469 cells, a type of normal liver cell line form mice (ATCC), were adopted in cell experiments. Firstly the toxicity test of glibenclamide was conducted. NCTC-1469 cells were pre-treated with different concentration of glibenclamide for 1 hour. Then cells viability was tested 24 hours later. As a result, cells viability decreased significantly at 500μM (Figure 3A; *P*<0.05). Accordingly 100μM of glibenclamide was selected as optimum concentration for subsequent experiments. Next we detected cell viability, potential of proliferation and apoptosis to evaluate cells radiosensitivity.

Cell viability and potential of proliferation were determined by CCK-8 assay and colony forming assay respectively. Upon different dose of IR the cells viability and colony forming rate decreased, meanwhile 1h pretreatment of glibenclamide partly rescued this reduction (Figure 3B, 3C, 3D, and 3E). Cell apoptosis rate is an important indicator for radiosensitivity because apoptosis is the main type of cell death under irradiation [14, 15]. In this study cells apoptosis rate were evaluated by Hoechst33342 staining and flow cytometry. IR dose and test time selection were based on the result in Figure 3B and Figure 3C that 48h after 8Gy of irradiation cells were detected apoptosis. Consequently irradiated NCTC-1469 cells were stained with extensively high-intensity of Hoechst33342, which indicated cell apoptosis due to pyknosis of nucleus. In contrast glibenclamide pretreatment significantly reduced the sum of high-intensity cells (positive rate of Heochst33342: 67.0±10.5% *VS* 36.3±3.5%; *P*=0.008; Figure 3F, 3G). Results of flow cytometry also demonstrated the remission of glibenclamide in radiation-induced apoptosis (apoptosis rate: 30.7±3.8% *VS* 15.4±4.1%; *P<*0.001; Figure 3H, 3I)

### Glibenclamide elevated membrane potential (MP) and activated reactive oxygen species (ROS)

**Figure 4:**
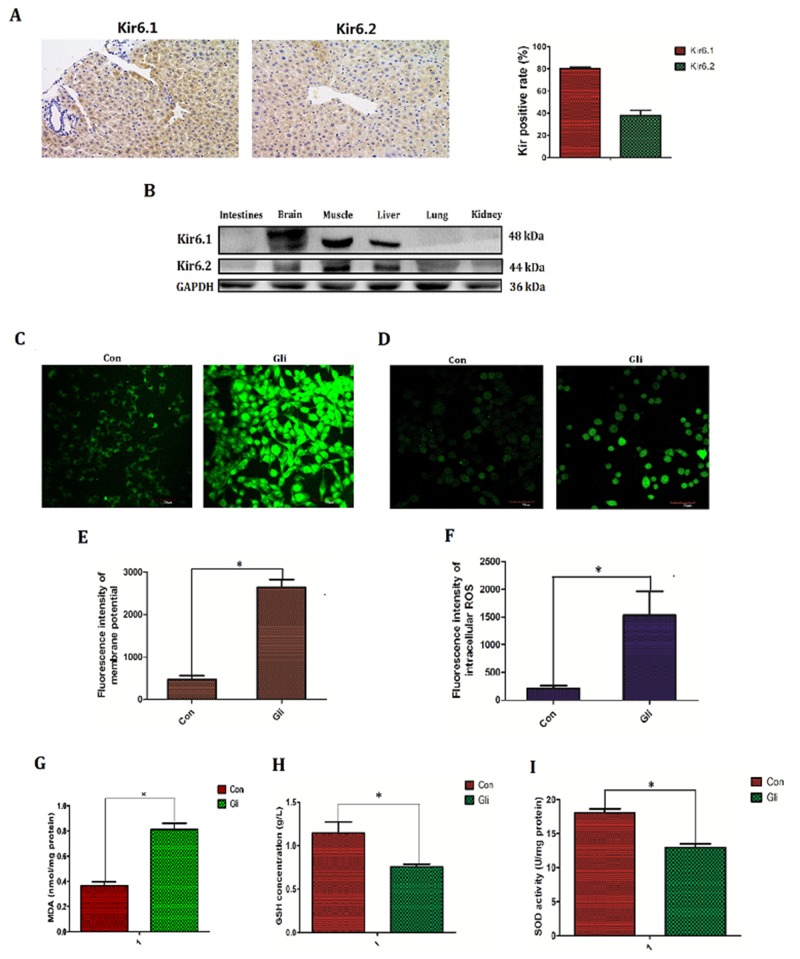
Glibenclamide elevated membrane potential and subsequently increased reactive oxygen species ATP-sensitive potassium channel (K_ATP_) is the target of glibenclamide and K_ATP_ consist of Kir6.1, Kir6.2, SUR1 and SUR2. The expression of Kir6.1 and Kir6.2 in hepatic tissue of mice (n=3) were tested by **(A)** immunohistochemical staining and **(B)** various tissues were detected by western blotting (n=3). 1 hour after treatment with glibenclamide (100μM) NCTC-1469 cells **(C, E)** were stained by DiBAC4(3) to test membrane potential (MP) and **(D, F)** were labeled by Reactive Oxygen Species Fluorogenic Probe to visualize ROS level. Simultaneously **(G)** the content of MDA was measured to indirectly demonstrate ROS level. **(H)** GSH concentration and **(I)** SOD activation were tested to reflect intracellular antioxidant ability. Data were presented as mean± SD. *P<0.05.

Except for the simulative effect on secretion of insulin, ATP-sensitive potassium channel (K_ATP_), the target of glibenclamide is also found to be associated with oxidative stress and generation of reactive oxygen species (ROS) though the exact conclusion remains to be controversial[11–12]. On the other hand increased ROS is generally taken for the main mediator to irradiation injury [16]. Thus in next study, we investigated if the protective effect of glibenclamide is associated with K_ATP_ and intracellular ROS. Firstly we verified the exact expression of K_ATP_ in hepatic tissue and other tissues. Results of immunohistochemical and western-blotting confirmed that K_ATP_ highly expressed in brain, muscle and liver tissues. In addition higher expression in Kir6.1 than Kir6.2 was observed in hepatic tissue of mice (Figure 4A, 4B).

Intracellular ROS level was detected by Reactive Oxygen Species Fluorogenic Probe and was observed with confocal microscopy. Besides, the content of MDA and activation of antioxidant enzyme including GSH, SOD were also tested. As a result glibenclamide group had higher flsorescence intensity (1530.28±413.26 *VS* 209.99±44.29; *P*<0.001; Figure 4D, 4F), increased content of MDA (0.81±0.11 *VS* 0.36±0.07; *P*<0.001; Figure 4G), decreased activation of GSH (0.75±0.07 *VS* 1.14±0.28; *P*=0.019; Figure 4H) and reduced content of SOD (12.96±1.27 *VS* 18.09±1.22; *P*<0.001; Figure 4I) indicating that glibenclamide lead to the elevation of intracellular ROS level.

Membrane potential (MP), especially the mitochondrial transmerabrane potential (MMP) is well known to regulate intracellular ROS level and is regulated by K_ATP_ [17, 18]. Thus MP was further detected by DiBAC4(3) staining. Consequently glibenclamide significantly increased intracellular membrane potential (MP) (2164.45±184.30 *VS* 469.60±88.32; *P*<0.001; Figure 4C, 4E).

### Glibenclamide activated Akt–NF-κB pathway via up-regulating ROS

Unexpectedly glibenclamide was verified to up-regulate intracellular ROS level. As ROS is the crucial mediator to radiation injury[6], we originally hypothesize that glibenclamide should reduce ROS to protect hepatocyte from radiation. Despite this perplexing result, some studies, indeed have reported that modest ROS could induce Akt and NF-κB activation to enhance cells survival [25–28]. Therefore we speculated that pre-treatment of glibenclamide might moderately up-regulate intracellular ROS level and subsequently activate Akt–NF-κB pathway to enhance hepatocyte survival against radiation.

**Figure 5:**
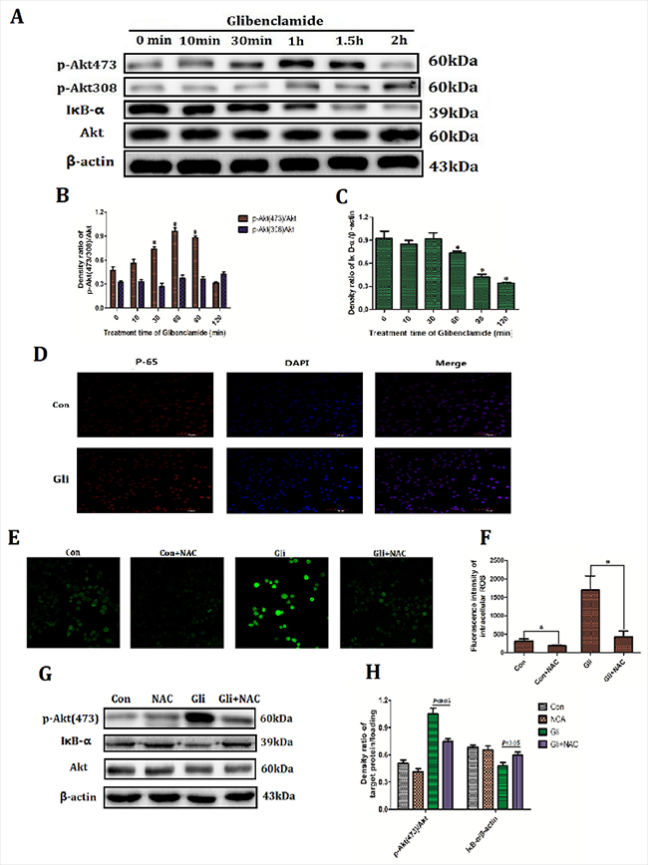
Glibenclamide activated Akt–NF-κB pathway via increased ROS NCTC-1469 cells were treated with glibenclamide (100μM) and then cells were harvested at 10min, 30min, 1 hour, 1.5 hour and 2 hour to extract proteins. **(A, B, C)** Expression of Akt and IκB were detected by western blotting and **(D)** translocation of p65 into cell nucleus was manifested by immunofluorescence. 1 hour after adding NAC (500μM) **(E, F)** the intracellular ROS level was marked by Reactive Oxygen Species Fluorogenic Probe and was visualized with confocal microscopy. **(G, H)** The expressions of Akt and IκB were detected by western blotting. n=3. Data were presented as mean± SD. *P<0.05 *VS* control group.

Firstly the relation between glibenclamide and activation of Akt–NF-κB pathway was investigated. Western blotting analysis manifested that glibenclamide phosphorylated Akt at 473 locus rather than 308 locus and reached the peak at 1 hour (Figure 5A, 5B). In the meantime IκB degraded (Figure 5A, 5C) and p65 transferred into cell nucleus (Figure 5D). These results confirmed an activation of Akt–NF-κB pathway form glibenclamide. Previous results (Figure 4) demonstrated that glibenclamide could elevate MP and subsequently up-regulated ROS level. In order to verify that the activated effect of glibenclamide on Akt–NF-κB pathway is due to increased ROS, we applied the N-acetylcysteine (NAC), a specific scavenger for ROS. As a result NAC eliminated ROS (Figure 5E, 5F) and simultaneously inhibited the activation of Akt–NF-κB pathway from glibenclamide (Figure 5G, 5H).

### Elimination of ROS wiped off the protective effect of glibenclamide on irradiated NCTC-1469 cells

**Figure 6:**
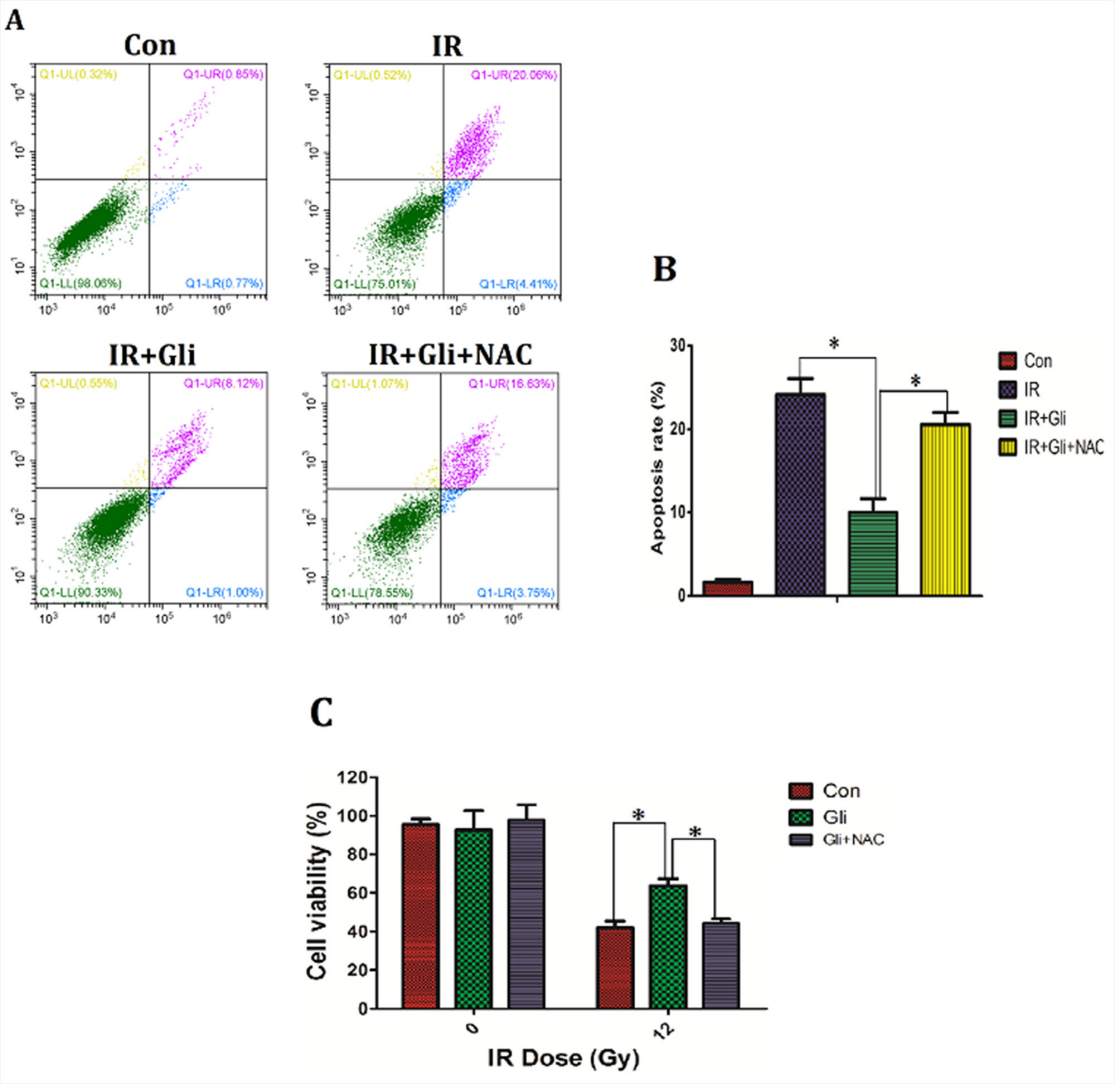
Elimination of ROS wiped off the protective effect of glibenclamide on irradiated NCTC-1469 cells NCTC-1469 cells were pre-treated with glibenclamide (100μM) or glibenclamide plus NAC (500μM) for 1 hour. After been replaced medium cells received single 8Gy of irradiation. **(C)** 24 h later the cells viability were tested by CCK-8 assay (n=3) and **(A, B)** 48 h later the apoptosis rate was detected by flow cytometry (n=3). Data were presented as mean± SD. *P<0.05.

Previous results had verified that glibenclamide up-regulated intracellular ROS level to activate Akt–NF-κB pathway. Given activation of Akt–NF-κB pathway has been verified to promote cells survival [27, 28], herein we further investigated if the up-regulation of ROS mediated the protection of glibenclamide on irradiated NCTC-1469 cells. By using NAC, the up-regulated ROS induced by glibenclamide was reduced (Figure 5E, 5F), and simultaneously eliminated the protective effect of glibenclamide on irradiated NCTC-1469 cells (Figure 6). Above results confirmed that glibenclamide protected irradiated hepatocyte by activating intracellular ROS.

### Effect of glibenclamide on hepatoma carcinoma cell

**Figure 7:**
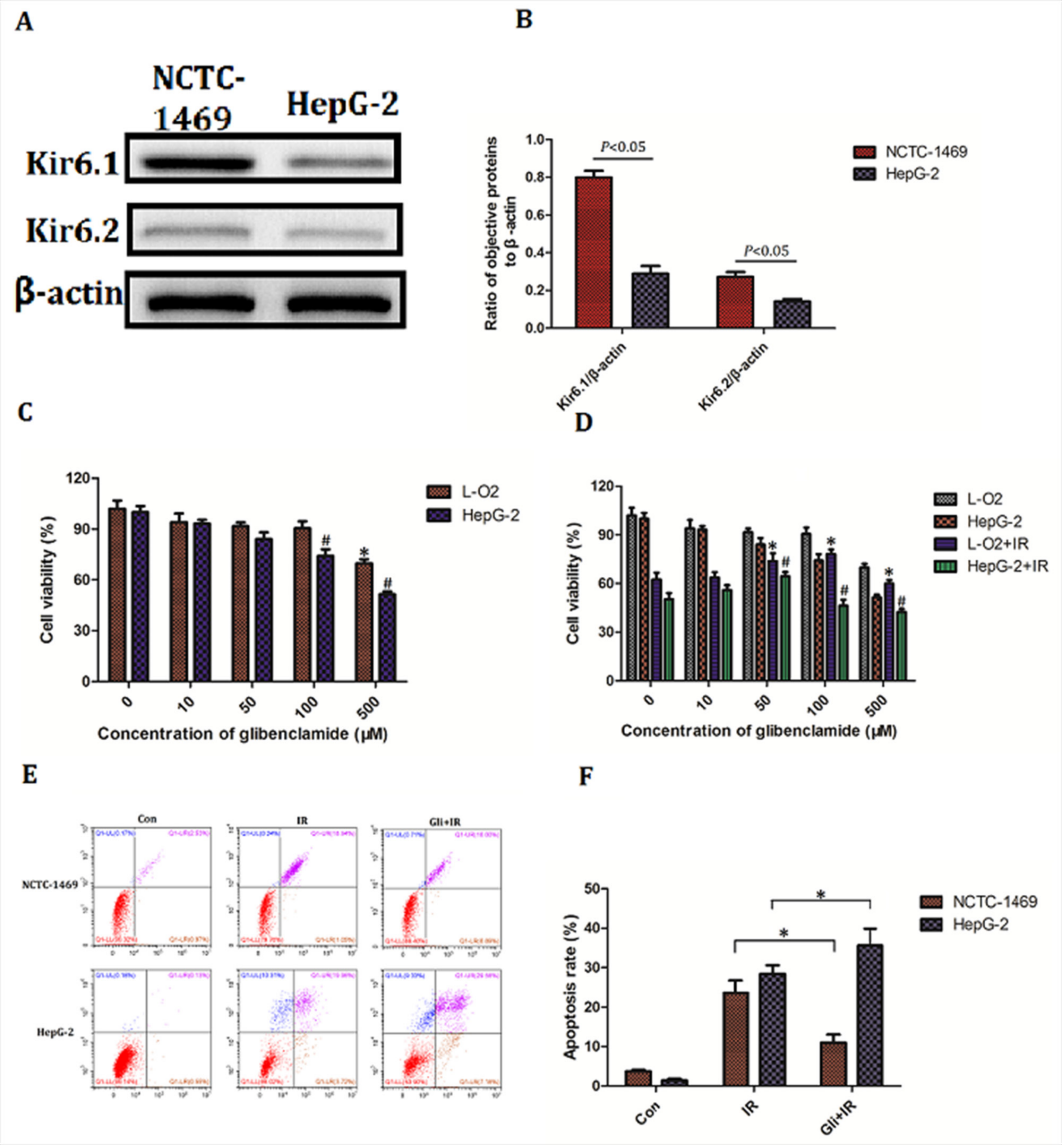
Effect of glibenclamide on hepatoma carcinoma cell **(A)** Human hepatic cell line L-O_2_ and human hepatoma cell line HepG-2 was harvested to detect the protein expression of Kir6.1 and Kir6.2 by western blotting. **(B)** Quantitative analysis were performed (n=3). **(C, D)** L-O2 and HepG-2 were treated with different concentration of glibenclamide for 1 hour. 24 hour after combining with/without IR the cells viability was determined by CCK-8 assay. **(E, F)** L-O2 and HepG-2 were pre-treated with 100μM glibenclamide for 1 hour. 48 hour after 8Gy of irradiation the cells apoptosis rate were tested by flow cytometry (n=3). Data were presented as mean± SD. *P<0.05 *VS* L-O2 at 0μM glibenclamide; #*P*<0.05 *VS* HepG-2 at 0μM glibenclamide.

Above results had confirmed that glibenclamide remitted acute radiation-induced liver disease and reduced radiosensitivity of hepatocyte. We then wondered the effect of glibenclamide on hepatoma carcinoma cell. Firstly the expression of K_ATP_ was investigated. Result of western blotting indicated a higher expression of Kir6.1 and Kir6.2 in human hepatic cell line L-O_2_ than in human hepatoma cell line HepG-2 (Figure 7A, 7B), suggesting hepatoma carcinoma cells have less expression of K_ATP_ than normal hepatic cells.

Secondly the toxicity of glibenclamide between L-O_2_ and HepG-2 was tested. With increased concentration of glibenclamide, cells viability of both L-O_2_ and HepG-2 decreased. However the significant toxic concentration was 500μM in L-O_2_ and 100μM in HepG-2, indicating that hepatoma carcinoma cells were more sensitive to glibenclamide (Figure 7C). Combining 8Gy of irradiation, glibenclamide at 100μM rescued cell viability of L-O_2_ but lead to inhibition in HepG-2 (Figure 7D). Meaningfully when concentration of glibenclamide reached to 500μM, both L-O_2_ and HepG-2 cell viability reduced significantly than radiation alone group. In addition result of flow cytometry also demonstrated that 100μM glibenclamide inhibited radiation-induced apoptosis in L-O_2_ but promotion in HepG-2 (Figure 7E, 7F).

## DISCUSSION

With the development of radiotherapy technology and exploitation of radioembolization the therapeutic selection for liver cancer obtains more chances though conventionally operative treatment has been applied for years.

However despite the therapeutic benefit, radiation induced liver disease (RILD) has become a rising concern. Generally, RILD contains two types, classical (patients without underlying liver disease) and nonclassical (patients with underlying liver disease). Patients with classical RILD presents as isolated elevation of alkaline phosphatase out of proportion to other liver enzymes, in contrast non-classical RILD is characterized as jaundice and markedly elevated serum transaminase [4, 12]. The course of RILD is complex and multistage, including early acute radiation-induced injury to hepatocyte and late fibrosis in interstitial tissue [19, 20]. In the early stage radiation leads to cells damage by directly breaking DNA strands and indirectly elevating reactive oxygen species (ROS) level [16]. The increased ROS extensively binds and damages biomacromolecules such as proteins and nucleic acid, which consequently leads to cellular death. On the other hand radiation could also stimulate immune cells and promote release of inflammatory factors including TGF-β, IL-6 and TNF-α. The activation of inflammatory response promotes fibroblasts transformation so that finally brings to hepatic fibrosis in the late stage [5]. Due to the great regenerative ability of hepatocyte, hepatic fibrosis is clinically seldom to occur but acute hepatic injury often cause severe outcome. In addition early acute hepatic injury is considered as central role in the development of RILD [20]. Therefore early hepatocyte protection is crucial for RILD therapy. Due to the complicacy of pathogenesis and fierceness of irradiation there is no effective and specific pharmacologic therapy for acute radiation induced-liver injury.

Glibenclamide is a classical and prevalent drug for diabetes since its production in 1957 [7]. It is a consensus that glibenclamide blocks ATP-sensitive potassium channel (K_ATP_) to induce the Ca^2+^ influx and subsequently increased release of insulin so that regulating the blood sugar level [7]. However recent studies demonstrated that except induction of intracellular Ca^2+^ glibenclamide regulated intracellular ROS by targeting K_ATP_ [8, 9]. Generally activation of K_ATP_ channel is believed to reduce endogenic ROS and blocking K_ATP_is regarded as an approach to increase ROS [10, 21]. However some studies come to opposite conclusion [11]. Despite the controversy glibenclamide is indeed involved in regulation of intracellular ROS level. As increased ROS is the main mediator to radiation injury. We accordingly speculated that the glibenclamide might influence the cell radiosensitivity. Thus in this study we investigated the effect of glibenclamide on acute radiation-induced liver injury and irradiated hepatocyte.

Firstly the acute radiation-induced model of BABL/C mice was established. After irradiation in hepatic region, livers and bloods of mice were taken to test pathology and measure serum transaminase respectively. Interestingly, the hepatic histopathology presented an extensively hepatocellular edema surrounding sublobular veins and local erythrocyte exudation under venous endodermis after irradiation but the serum transaminase including ALT, AST didn't indicate significantly change. On the other hand, the serum content of ALP reached to marked elevation after irradiation. This result is in line with previous studies, which described an isolated elevation in alkaline phosphatase out of proportion to other liver enzymes [4]. It may be explained that cellular and matrical edema are the main denaturation of acute radiation-induced injury, which is limited to damage cells and subsequently released transaminase into serum. Except hepatocellular edema, the histo-pathological images also showed sublobular veins injury. In fact the vascular endothelial cell is more sensitive to irradiation than the liver parenchyma cells, so some studies regarded the vascular injury as the primary and critical link to radiation-induced liver. With the vascular injury, liver parenchyma cells are gradually exposed to ischemic microenvironment so that develop to edema or other lesions.

Using this acute radiation-induced liver injury model we tested if the glibenclamide play a role. As a result the pre-treatment of glibenclamide mitigated acute radiation-induced liver injury of mice, which performed as regression of hepatocellular edema and reduction of hepatic sinusoid. To further verify this protective effect, we then tested the effect of glibenclamide on NCTC-1469 cells, a normal liver cells line from mice. According with *in vivo* results, glibenclamide could reduce the NCTC-1469 cells radiosensitivity. Thus we confirmed that the protective effect of glibenclamide on hepatic tissue and hepatocyte. These results, to our knowledge, are firstly reported, though glibenclamide have been applied and studied for many years.

**Figure 8:**
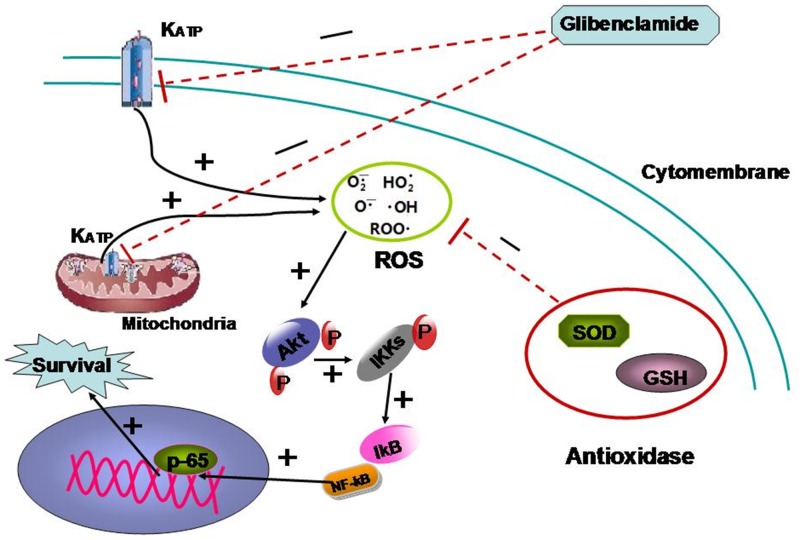
The mechanism summary of glibenclamide protecting irradiated hepatocyte

It was interesting and surprising that a classic drug for diabetes mellitus might be a radioprotectant and the mechanism involved should be researched in-depth. Concerning the specific mechanism we focused on the association of K_ATP_ and intracellular ROS. K_ATP_ is believed to be associated with intracellular ROS level by affecting membrane potential (MP) [17, 18] As glibenclamide could specifically target K_ATP_, so glibenclamide is speculated to affect irradiated cells by changing intracellular ROS level which is generally considered as the main mediator to radiation-induced injury [6]. Unexpectedly, glibenclamide elevated MP and subsequently increased intracellular ROS. It is well known that radiation induced increase of ROS to damage biomacromolecule and lead to apoptosis [6]. We originally hypothesize that glibenclamide should reduce ROS to protect hepatocyte from radiation. Despite this perplexing result, some studies, indeed have reported that moderate prestimulation of ROS could activate Akt pathway to enhance cells survival [25–28]. Thus we speculated that pre-treatment of glibenclamide stimulated Akt-NF-κB pathway and it was the stimulation that could protect cells from drastic ROS induced by radiation. Accordantly western blotting and immunofluorescence detection revealed that glibenclamide phosphorylated Akt as well as promoted the transfer of p65 into cell nucleus. With using NAC, a scavenger for ROS, the stimulated response was eliminated. What's more NAC also eliminated the protective effect of glibenclamide on irradiated cells. Above results confirmed the mediated role of ROS in the effect of glibenclamide. The mechanism is summarized as Figure 8.

Having confirmed the protection of glibenclamide on hepatocyte, we then wondered if glibenclamide play a role in hepatoma carcinoma cell. Our results demonstrated that beyond specific concentration (500μM for L-O2, 100μM for HepG-2), glibenclamide could exert inhibition on both L-O2 and HepG-2. This result is accordance with other studies [22, 23] that glibenclamide showed antitumor effect though so far there is no report on liver cancer. However the toxic concentration of L-O2 is significantly less than HepG-2. HepG-2, as a kind of hepatoma carcinoma cell, naturally restores high level of intracellular ROS than normal hepatocyte [24]. Hence HepG-2 may represent lower tolerance to glibenclamide given glibenclamide is a source of ROS and play antitumor effect. Combining with irradiation, low concentration of glibenclamide (50μM) protected both L-O2 and HepG-2, but at 100μM, the concentration of still protecting L-O2 in contrast promoted radiation-induced apoptosis of HepG-2. This result indicated the difference of applied concentration of glibenclamide between hepatocyte and hepatoma carcinoma cell, suggesting that at a dose range glibenclamide may remit acute radiation-induced liver injury as well as inhibit liver cancer. Anyway the exact conclusion remains to be confirmed by more well-designed experiments in the future.

As far as we know this study is the first investigation that revealed a protective effect of glibenclamide on acute radiation-induced liver injury. Though the advanced stimulation of ROS was verified as the critical mediator there were still limitations to know if ROS could directly stimulated Akt and specific combination mode still remained unclear. Above all things our results indicated that glibenclamide prevents acute radiation-induced liver injury via up-regulating intracellular ROS and subsequently activating Akt–NF-κB pathway. As the specific highly expression of K_ATP_ channel in liver tissues and explicit toxicity of glibenclamide, we suggested the clinical potential of glibenclamide in the therapy of acute radiation-induced liver injury.

## MATERIALS AND METHODS

### Mice

BALB/C male mice, 5-6 weeks of old, was from Shanghai Ling Chang biological technology co., LTD. Mice was kept in Specific Pathogen-Free (SPF) facility for all experiments. All the experiments associated with mice obtained approval of the Laboratory Animal Center of the Second Military Medical University, Shanghai.

### Cell and cell culture

NCTC1469 cells (a type of normal mice liver cell line), L-O2 cells (human hepatic cell line) and HepG-2 (human hepatoma cell line) were from American Type Culture Collection, Manassas, VA, USA (ATCC). Cells were cultured using DMEM Medium (PAA, Austria) containing 10% fetal calf serum (PAA, Austria) and were incubated under 5% CO_2_ humidified chamber at 37°C. Cells were observed with microscope every day and medium was replaced intermittently. Finally the cells at logarithmic phase were harvested to use for experiments.

### Irradiation of mice liver and liver cell line

^60^Co source in the radiation center (Faculty of Naval Medicine, Second Military Medical University, China) was used for irradiation. In order to confine irradiation only in the liver of mice without damaging other tissues, we had the leads shielded other tissues so that only the liver was exposed to the beam. All the livers of mice received a single dose of 30 Gy at a dose rate of 1 Gy/min. Cell lines received a single dose of 6, 8, 12 Gy.

### Biochemical analysis of mice serum

The hepatic function of mice was determined by the serum level of alanine aminotransferase (ALT), aspartate aminotransferase (AST) and alkaline phosphatase (ALP). These measurements were made in “angel” pet hospital in Shanghai.

### Liver histological analysis and quantitative score

The livers of mice were taken according to study design, and then the liver specimens were dehydrated, embedded in paraffin and then sectioned into at least 7 slices with the thickness of 5 μm. After the slices were dewaxed in xylene and rehydrated by exposure to graded ethanols, tissue sections were stained with Haematoxylin-Eosin. Histological images were observed and were photographed with microscope. Slides were manually scored in a blinded fashion for degree of hepatocellular vacuolation. The vacuolar differences, when present, were primarily from periportal to midzonal hepatocytes; thus, scoring was based on the average of three periportal images per liver section. For each image, 100 hepatocytes were counted and given a score corresponding to the severity of the vacuolation: low, medium, or high. Low vacuolation was characterized by mild cytoplasmic vacuolation consistent with glycogen accumulation. Medium to high vacuolar scores corresponded to hepatocytes that were moderately to markedly enlarged due to dilation of the cytoplasm by clear.

Space and poorly defined clear vacuoles [12]. Histological results had been certified by a pathologist MD Qian Ma from Department of Pathology, Ruijin Hospital, Shanghai Jiaotong University School of Medicine, Shanghai 200025, P.R. China.

### TUNEL assay and evaluation

dUTP nick-end labeling (TUNEL) assay was used to determine apoptosis in liver tissues. Paraffin-embedded sections (3 μm thick) of liver tissues were fixed in paraformaldehyde solution (3% in phosphate-buffered saline [PBS], pH 7.4) for 5 min at room temperature. The fixed sections were washed with PBS for 5 min at room temperature and then treated with proteinase K in HCl (0.01 N, pH 2.0) for 15 min at room temperature. Then 110 μL of the TUNEL reaction mixture was added to each section, which was then coverslipped and incubated in a humidified chamber at 37°C for 60 min. Subsequently, 65 μL antidigoxigenin conjugate solution was applied to each specimen and the sections were incubated for 30 min at room temperature. The reaction was carried out by adding 75 μL of a peroxidase substrate solution to the sections for 6 min at room temperature. The slides were washed with PBS and mounted in 0.5% methyl green. TUNEL was quantified by counting positive hepatocyte nuclei (brown) per 400x field. A total of 5×400x fields were scored per liver, and means of these scores were calculated for further statistical analysis [13].

### Electron microscopic (EM)

Removed liver tissues of mice were fixed with 2.5% glutaraldehyde in 0.1 mol/L phosphate buffer (pH 7.4), followed by 1% OsO4. Slices were dehydrated and then were stained with uranyl acetate. Finally the tissues were observed by electron microscope.

### CCK-8 assay and clonogenic survival

CCK-8 assay (Cell Counting Kit-8; Dojindo, Kumamoto, Japan) is an improved method for detecting cell viability, which is in principle similar to MTT assay. Briefly cells were planked in the 96-well plates as the density of 5000 cells per well before irradiation. 24 hours after irradiation, cells were co-incubated with CCK-8 reagent. 2 hours later the OD values were measured by microplate reader. Clonogenic survival was performed to assess the potential of cell proliferation. Firstly cells were planked in 6-well plate and the planked cell number was based on the previous irradiated dose-survival curve. After irradiation cells were continually cultured under 5% CO_2_ humidified chamber at 37°C for 10 days. Finally the clones were stained with crystal violet hydrate solution containing 5% formaldehyde and were counted.

### Measurement of intracellular GSH, SOD, MDA and ROS level

The GSH, ROS, and MDA level of NCTC1469 cells were measured with GSH, SOD and MDA assay kit respectively (Cat. No S0053, S0109, S0131; Beyotime; China). The intracellular ROS was detected by Reactive Oxygen Species Fluorogenic Probe (Cat. No S0033; Beyotime; China) and was observed with confocal microscopy.

### Western blot analysis

NCTC1469 cells and liver primary tissues were collected after treatment. Then cells were resuspended in PBS and washed for 3 times. Collected in extraction buffer (1% Triton X-100,0.5% sodium deoxycholate, 20mM Tris-HCl, pH 7.5, 12mMglycero-phosphate, 150mM NaCl, 5mM EGTA, 10mM NaF, 3mM dithiothreitol, 1mM sodium orthovanadate, 1mM phenylmethylsulfonyl fluoride, 20g/ml aprotinin), cells were incubated on ice for 3×10 minutes, and lysate was centrifuged (12,000g for 15 minutes at 4°C) to obtain the supernatant containing protein. Equal amount of proteins was separated on a 10% SDS-PAGE gel and transferred to nitrocellulose membranes. 5% low-fat milk was used to block the membranes for 2 hours then the membranes were washed 3 times with TBST for 10mins. The membranes were probed overnight at 4°C with antibodies recognizing the following antigens: Kir6.1 (1:1000); Kir6.2 (1:1000); Akt1 (1:1000), p-Akt(308) (1:1000), IκB-α (1:1000). Kir6.1, Kir6.2 antibodies were purchased from Abcam Corporation, and other antibodies were obtained from Cellsignal Corporation. The antibody-antigen complex was detected using horseradish peroxidase-conjugated secondary antibody. Peroxidase labeling was visualized with enhanced chemiluminescencelabeling using an ECL Western blotting detection system (Thermo, USA)

### Statistical analysis

We used one-way ANOVA followed by Scheffe's test to analyse any difference between control and irradiated cultures at each single culture time-point. All reported values are expressed as means ± SEM for each experiment. The number of samples is indicated in the description of each experiment. P < 0.05 was considered statistically significant.
